# “If it’s not Iron it’s Iron f*cking biggest Ironman”: personal trainers’s views on health norms, orthorexia and deviant behaviours

**DOI:** 10.1080/17482631.2017.1364602

**Published:** 2017-08-21

**Authors:** Linn Håman, Eva-Carin Lindgren, Hillevi Prell

**Affiliations:** ^a^ School of Health and Welfare, Halmstad University, Halmstad, Sweden; ^b^ Department of Food and Nutrition, and Sport Science, University of Gothenburg, Gothenburg, Sweden

**Keywords:** Diet, disordered eating, exercise dependence, fitness culture, focus groups, gym

## Abstract

Orthorexia nervosa (ON) describes a pathological obsession with healthy eating to avoid ill health. In the Swedish context, ON is also understood in terms of unhealthy exercise. Fitness gyms are popular health-promoting places, but exercise-related problems, disordered eating and ON-like behaviour are increasing. Personal trainers (PTs) play an important role in detecting unhealthy behaviours. The aim of the present study was to illuminate PTs’ understandings of healthy and unhealthy exercise and eating behaviours in relation to orthorexia nervosa in a fitness gym context. Five focus groups with 14 PTs were conducted. These were analysed using interpretative qualitative content analysis and Becker’s model “Kinds of Deviance.” In contrast to PTs’ health norms (practicing balanced behaviours and contributing to well-being), ON was expressed mainly in terms of exercise behaviour and as being excessive and in total control. The PTs maintain that extreme behaviours are legitimized by an aggressive exercise trend in society and that they fear to falsely accuse clients of being pathological. Certain sport contexts (bodybuilding, fitness competitions and elite sports) and specific groups (fitness professionals) contribute to complicating PTs’ negotiations due to a competition, performance and/or profession norm, making it difficult to determine whether or not to intervene.

## Introduction

Since the 1970s a greater individual responsibility for achieving health has been emphasized, framing it as a moral obligation—the ideology of “healthism” (Crawford, 1980; Lupton, 1995). According to this ideology, health is considered to be a goal in itself, not a means for attaining other goals in life (cf. WHO, 1986). Health should be maintained by discipline and self-control and by the individual’s own effort (Crawford, 1980). In recent years scholars have argued that societal health ideals have become more aggressive because of the commercialization of the fitness culture (Jönsson, 2009). However, what is regarded as healthy is influenced by different health norms that are negotiated and change over time and that vary in different contexts (cf. Becker, 1963; Pelters, 2012). The view of health in this study is based on a practical definition that includes subjective experiences of health that is contextual, more specifically: “Health is the experience of physical and psychological well-being” (Card, 2017, p. 131). This health definition also states that health occurs on a continuum and that absence of disease is not necessary for a feeling of good health (Card, 2017).

In a time with healthy ideals connected to moral imperatives (Crawford, 1980; Lee & Macdonald, 2010; Lupton, 1995) and the medicalization of social problems (Vanderycken, 2011), the term orthorexia nervosa (ON) was first proposed by Bratman (1997) (Dunn & Bratman, 2016). Orthorexia nervosa is defined as “a pathological obsession with healthful eating” (Dunn & Bratman, 2016, p. 12) in order to pursue health and to avoid ill health and disease (for a current review see Dunn & Bratman, 2016). What is perceived as healthy among individuals who are considered to have ON is based on subjective criteria (Bratman & Knight, 2000). However, research covering ON is still limited (e.g., Håman, Barker-Ruchti, Patriksson, & Lindgren, 2015) and diagnostic criteria have only recently been proposed (Dunn & Bratman, 2016). Today, ON is not an accepted diagnostic category, that is, it is not included in the diagnostic manuals *DSM-5* or *ICD-10* (Varga, Dukay-Szabó, Túry, & Van Furth, 2013). Research has shown that ON shares many characteristics with diagnosed eating disorders (ED) and Obsessive Compulsive Disorder (Koven & Abry, 2015), and ED professionals suggest that ON deserves more attention in research and clinical practice (Vanderycken, 2011). In a Swedish context, ON is understood in terms of unhealthy exercise, and it has been linked to fitness gyms and emerging aggressive health and exercise trends (Håman, Barker-Ruchti, Patriksson, & Lindgren, 2016). Indeed, gyms are a central place for taking care of one’s own health (Andreasson & Johansson, 2014, 2015a). At the same time, they are a place where unhealthy and destructive eating and exercise behaviours have been reported (Bratland-Sanda & Sundgot-Borgen, 2015; Hale, Roth, DeLong, & Briggs, 2010; Höglund & Normén, 2002). In gym settings PTs play an important role in dealing with different health-related behaviours (Bratland-Sanda & Sundgot-Borgen, 2015).

Today, PT is one of the fastest growing career options in Western societies (Nygård, Gjølme, & Leirdal, 2015). Personal trainers plan and conduct individualized exercise programmes with a client and have competence in, for instance, client analysis, body tests and nutrition (Nygård et al., 2015). The education required to become a licensed PT varies, but is in general limited given the complexity of the role and the high demand of responsibility, knowledge and competence. Therefore, a great responsibility falls on PTs, and practice-based learning is crucial. Several norms regarding health, exercise and diet prevail in gym settings, which influence PTs’ learning and ideas (Andreasson & Johansson, 2015b). For instance, strict diets and high volumes of exercise constitute some of the existing norms at the gym (Andreasson & Johansson, 2015a; Bratland-Sanda & Sundgot-Borgen, 2015). These norms can complicate work to promote health and to detect and handle problematic behaviours related to diet and exercise (Bratland-Sanda & Sundgot-Borgen, 2015). In addition, no consensus exists in the scientific literature regarding when exercise becomes excessive or compulsive (Bratland-Sanda & Sundgot-Borgen, 2015; Meyer, Taranis, Goodwin, & Haycraft, 2011). Thus, PTs’ views concerning the border between healthy and unhealthy behaviour are relevant to further examine since they might meet clients with varying behaviours that influence health. For instance, almost 50% of fitness instructors reported disordered eating concerns among one or more members (Bratland-Sanda & Sundgot-Borgen, 2015). How PTs discuss and draw the boundary line between healthy and unhealthy behaviours is crucial to how they respond to members. There are several circumstances and health norms that can influence and complicate PTs’ discrimination between these behaviours (cf. Becker, 1963). In this study, behaviours that the PTs associate with ON will constitute a base for unhealthy behaviours. The focus on ON is a way of delimiting the material and also focusing on a contemporary concept that has received increased attention in Sweden (Håman et al., 2016). The aim of the present study is to illuminate personal trainers’ understandings of healthy and unhealthy eating and exercise behaviours in relation to ON in a fitness gym context. Our research questions, therefore, are: (1) How do personal trainers understand ON with reference to deviant behaviours and health norms? (2) How do personal trainers negotiate the borderland between healthy and unhealthy exercise and eating behaviours?

### Health norms and deviant behaviours

**Figure 1.**
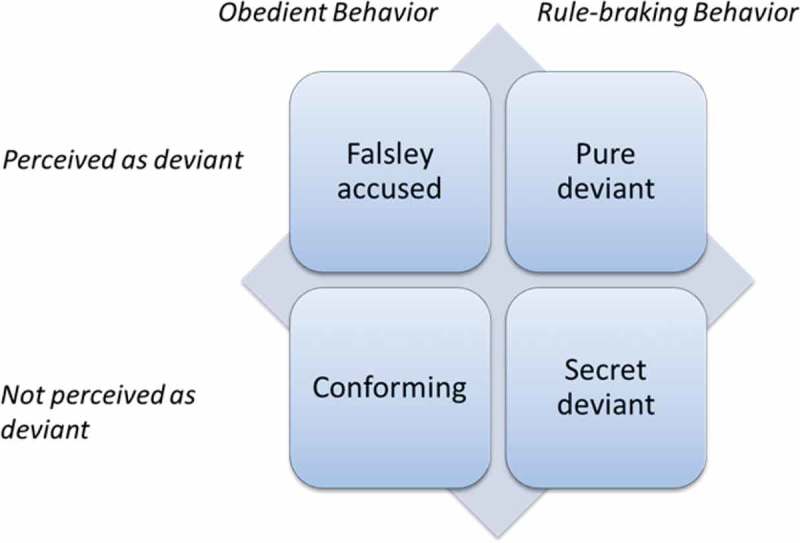
Kinds of deviance: a sequential model (Becker, 1963, p. 20).

Becker´s (1963) sequential model “Kinds of Deviance” (see Figure 1) was employed to analyse and interpret PTs’ understandings. The model involves discrimination between different types of deviance that enables understanding of how deviant behaviours occur and originate in one particular context. The model is called sequential since it acknowledges that notions of deviant behaviours will change over time. Personal trainers’ boundary lines between healthy and unhealthy behaviours, including ON, can be influenced by societal and social norms about health. Norms are rules and expectations by which members of particular groups and contexts are guided (Becker, 1963). As such, norms are created by particular groups and vary throughout different cultures and contexts. For instance, deviant behaviours can break the norms in one sociocultural context but may be regarded as legitimate within another context. Behaviours that transgress accepted health norms in the gym setting will be regarded as deviant. Health norms in gym settings are created and constantly negotiated by participants and fitness professionals (cf. Becker, 1963). However, the creation and negotiation of norms cannot be seen as an isolated process within specific settings. Norms are part of a larger societal and cultural context in which they are modified, produced and reproduced (Becker, 1963).

In the model (Figure 1), four claims create a set of quadrants of different kinds of deviance as perceived by the PTs—pure deviant, conforming, falsely accused and secret deviant. The first quadrant, pure deviant, involves rule-breaking, non-conforming behaviours that are perceived as deviant. The second quadrant, conforming, refers to behaviours that obey rules (i.e., conform to accepted norms) and also are regarded as obedient among others. Falsely accused, the third quadrant, includes behaviours that are obedient but perceived as deviant. The last quadrant, Secret deviant, refers to behaviours that are rule-breaking, although others do not react to them as deviant.

## Methods

Five focus-group discussions (FGDs) were conducted with a total of 14 PTs. These were analysed by means of interpretative qualitative content analysis (Krippendorff, 2013). This approach is founded on the notion that individuals define meaning and understanding that are embedded in social interaction within their context. Indeed, the intent is to create understanding of these meanings and contents (Thorne, Kirkham, & O’Flynn-Magee, 2004).

### Sample and procedure

The PTs were recruited through purposive sampling (see Oliver, 2006). Inclusion criteria were that they were currently working as PT and having heard about ON. When the fitness gyms and PTs were selected, the aim was to gain as wide a variation as possible concerning gender, fitness gym locations regarding socioeconomic factors, association and commercial form. Local managers at 13 registered fitness gyms (3 local and 10 included in gym chains) were contacted and informed about the study and asked for permission to contact PTs at their gyms in the area of western Sweden. In all 10 gyms (2 local and 8 included in gym chains) gave their permission. An email including information about the study was sent to 30 PTs. This recruitment did not generate enough participants. Therefore, the email was sent to all PTs at each fitness gym. A reminder was thereafter sent to specifically selected PTs to gain a wider variation. The 14 PTs in the five focus groups (8 women and 6 men) were aged 23–47 years. Personal trainers within FGDs 1 to 4 were employed by the hour, whereas the PTs in FGD 5 held a permanent position. All of them had a nutritional advisory role. Characteristics of the FGDs are outlined in Table 1. Focus-group discussions 1 and 5 were “mini groups”, with four PTs in each (cf. Roller & Lavrakas, 2015). Due to difficulties in the recruitment process, FGDs 2–4 only contained two PTs in each group (see Table 1). Focus-group discussions of this size are termed a “dyad” (Roller & Lavrakas, 2015). Within FGD 1, all PTs had met before; two of them were co-workers at the same gym; the PTs were also co-workers in FGD 5. In the other FGDs the PTs had not met before.

**Table 1.** Focus groups included in the study.

Focus-group discussion	Participants (*n* [gender])	Age range (years)	Work experience as a personal trainer (years)	Number of hours spent at the gym per week	Length of personal trainer education	Length of continuing professional learning *	Bachelor degree in a field relevant for a personal trainer (*n*)**	Duration of focus-group discussion (minutes)
1	4 (3 women, 1 man)	24–43	1.5–4	10–70	2 weeks −6 months	2 days–3 weeks	1	124
2	2 (1 woman, 1 man)	23–26	1–5	15–40	2 weeks- 1.5 years	3 –6 months	0	73
3	2 (2 women)	33–40	0.5–1	30–40	2 months-1 year	3 days	1	103
4	2 (1 woman, 1 man)	31–35	1–12	20–28	5 weeks- 5 months	1 month	0	80
5	4 (1 woman, 3 men)	23–47	1.5–15	28–40	2 weeks- 3 years	1–11 days	4	107

* Compared with Coach Learning and Development (Cushion et al., 2010).

**For example Sport science and Physiotherapy.

### Focus group discussions

Focus-group discussions were employed since they catch the social interaction when individuals create meaning together (cf. Roller & Lavrakas, 2015). The first author acted as a moderator, the last author was an observer in FGDs 1–4, and another researcher was an observer in FGD 5. The FGDs were conducted in conference rooms at two different universities. The PTs were asked to read and sign consent forms acknowledging that they understood their rights and the voluntary nature of participation. They were also informed that: (1) the information provided by PTs and about their employers would be kept confidential and (2) the FGDs would only be used for research purposes. The Swedish Research Council’s ethical principles were followed, and the study is therefore in compliance with the applicable ethical rules (HSFR, 1991).

A thematic semi-structured interview guide was developed and tested by university students in a pilot study. At the beginning of each FGD discussion we explained that all statements would remain within the group, there were no right or wrong answers and all thoughts and opinions were important. Material to stimulate discussions was also used. Follow-up and probing questions were used throughout the discussions. After the FGDs, the PTs completed a one-page questionnaire for background data. In this questionnaire, the question regarding gender was followed by an open answer option in which the PTs had the opportunity to write which gender they identified with. The FGDs lasted 73–124 min. All FGDs were audio recorded and transcribed verbatim. To ensure confidentiality, PTs have been given pseudonyms by us. The pseudonyms were assigned based on which gender the PTs identified with.

### Data analysis

An interpretative qualitative content analysis was applied (Krippendorff, 2013). The analysis allowed interpretation of how PTs express ON with reference to deviant behaviours and health norms and how PTs negotiate the borderland between healthy and unhealthy exercise and eating behaviours. The analysis was conducted in two phases. The first phase was inductive and carried out as follows: (1) the transcripts were read several times to obtain an overall impression; (2) the transcripts were re-read to select meaning units (i.e., segments of conversations or single quotes) that responded to the aim; (3) the meaning units were coded into keywords or key phrases that reflected the content; (4) on the basis of similarities and differences, the keywords or key phrases were compared, sorted and arranged into tentative categories. Thereafter, the tentative categories were reviewed and discussed several times and then revised and encoded into six categories: (1) excessive and (2) controlling behaviour, (3) balanced behaviour, (4) contributing to well-being, (5) extreme behaviour and (6) healthy behaviour with an unhealthy mind. In the next phase, the analysis was more deductive. That is, the four types of deviant behaviour identified by Becker (1963) (pure, conforming, falsely accused and secret) were interpreted to fit the PTs’ understanding of deviant behaviour: (1) pure deviant behaviour relating to *excessive* and *controlling behaviour;* (2) conforming behaviour relating to *balanced behaviour* and *contributing to well-being;* (3) falsely accused behaviour relating to *extreme behaviours* as a consequence of an aggressive exercise trend; (4) secret deviant behaviour relating to *healthy behaviour with an unhealthy mind*. From the initial analysis we were able to recognize three categories (*fitness and bodybuilding competitions*, *elite sports* and *fitness professionals*) that governed PT negotiations concerning the borderland between healthy and unhealthy exercise and eating behaviours.

## Findings

### Pure deviant behaviours

Pure deviant exercise and eating behaviours are expressed as *excessive* and *controlling behaviour*. In PTs’ views, a few clients exhibited excessive exercise and dietary behaviours that they referred to as ON. Thus, behaviours associated with ON represent their examples of a completely unhealthy approach. Excessive behaviours are described in terms of being extreme, addictive and obsessive. In practice, excessive exercise refers to high frequency, duration and intensity, whereas excessive eating behaviours refer to ingesting food on a strict regular basis with an excessive focus on food quality and eating clean (e.g., only eating protein-rich foods):

Camilla: She [a girl at the gym] ran hard, first on the treadmill for like three hours and then did strength training and after that she maybe ate a bit, but she was at the gym for like six hours.

Linnea: Yes, you clearly have seen these kinds of people.

Camilla: She [the girl at the gym] was like totally crazy.

Linnea: Oh, now this person is back again.

Camilla: And then, you noticed her, like, “pumping iron” again…y’ know, it must be, if I’m thinking about orthorexia, she would be my prime example. (FGD 3)

The PTs, however, emphasize excessive exercise as the main characteristic of ON and that this is the most common way people in general understand ON. These excessive behaviours completely cross the healthy line regarding exercise and diet. They visibly break PTs’ health norms, since they negatively influence the clients’ lives by becoming all-consuming and contributing to the sacrifice of social activity.

To be in total control is perceived as a deviant behaviour. Unhealthy behaviours expressed as ON involve an extreme need to control food quality, such as scrutinizing the table of contents, but also restricting the intake of carbohydrates and fat, as well as counting calories. The control issue is so strong that the individual perceived to have ON cannot deviate from the plan (e.g., eating something other than he/she planned or missing an exercise session). In addition, people are perceived to exercise even though they, for example, have an infection, or they cannot rest for a week or go on vacation without exercising:

Moderator: Can you describe, what is orthorexia?

Jonas: [describes how he understands the characteristics of orthorexia]…you feel that you must work out to be in control. You have to have full control regarding both diet and maybe exercise, but the question is what is the difference between [a professional athlete and an everyday person]? Sure, the athlete has an aim and a goal, but they must also have full control of their diet and exercise….

Moderator: Mmm

Maja: [describing an individual she considers to have orthorexia]…if I [do not exercise a lot], I will get into the worst state of anxiety or feel very bad—it all becomes so controlled, it becomes too much….Sure, you can work out a lot, but you need to be able to relax for a week and take it easy and not get anxious about it, this is where the border for orthorexia occurs, you could say. (FGD 4)

Thus, the need for control has actually spun out of control, since the individuals are no longer perceived to be able to balance eating, exercise and recovery.

### Conforming behaviour

Conforming behaviours refer to healthy behaviours, which are expressed in terms of *balanced behaviours* and *contributing to well-being*. Balance refers to eating the right amount, exercising regularly and allowing oneself to recover. Personal trainers emphasize the importance of achieving a good balance between eating, exercise and recovery on an individual level, which depends on individual goals and abilities as well as genetic disposition. However, excessive exercise and eating behaviours can be accepted as balanced behaviour among some clients, as long as they have fun, are free from injury, have a social life and do not feel bad about it:

Carolina: …I would say that healthy exercise is very much an individual thing.…some people maybe want to work out two-three days a week, and if they feel good doing that, it is healthy. But there are also people who work out two times a day, seven days a week.…I think it is entirely dependent on the individual and on how they live. As long as it [the amount of exercise] doesn’t affect your social life…that the body still feels well and that you don’t get worn out and become worse, than it [the exercise] doesn’t become so healthy and if you carry on, get pain and hurt yourself easier, then it doesn’t become healthy either. You might say that healthy exercise involves being free from injury and having a social life.

Tobias: Yes, I agree, I would say the same as you….It is on an individual level because everybody have different genetic dispositions. It means some individuals can work out harder, whereas others might get injured by the same exercise program…. (FGD 2)

Thus, PTs had difficulty in determining the healthy amount of exercise because of individual differences. Instead, all exercise behaviours that contribute to a healthy body and mind as well as a feeling of well-being were brought to the fore regarding healthy behaviours.

### Falsely accused behaviours

Falsely accused refers to *extreme behaviours* as a consequence of an aggressive exercise trend. This trend is perceived by the PTs to have emerged in Sweden in the past few years and involves a culture with exercise and dietary behaviours approaching pure deviant behaviours. For instance, individuals exhibit more extreme control over what they eat, have increased limits of what is classified as moderate exercise and take part in more extreme competitions (e.g., Ironman Triathlon, marathons and ultra-races). Thus, this trend contributes to push the boundary lines of health norms regarding conformal healthy behaviours to a more extreme level, since unhealthy behaviours become normalized and socially accepted:

Maria: I believe that it [extreme exercise and healthy eating] is more undisclosed now than five years ago. Now it is OK to increase the dose and be more controlled, because individuals can almost do it [exercise extremely and regulate the diet] in a group, or through Facebook or through Instagram….People can even get likes [on Facebook and Instagram] for this, and they can meet people in the gym doing exactly the same thing….And then they can show it off and they can check their calories, they can eat their cottage cheese and their cod and all that, without anyone saying anything because everybody does it. There has been a huge difference over the last five years [the emerging exercise trend] it’s just like, poof! [makes explosion noise]. It is the same thing with these big races, if it’s not *Iron*, it´s *Iron fucking biggest Ironman* [Triathlon race].

Andreas: Yes, yes [laughing].

Maria: It [the emerging exercise trend] gets harder and harder, it will get bigger and bigger, there will be more and more and it is OK to do it, because everybody already does it.

Andreas: Yes, yes.

Maria: …nowadays it is socially accepted to do this, like it is just accepted in society. (FGD 5)

Thus, the PTs express that this trend contributes to complicating their determination of a client exhibiting unhealthy behaviours, since extreme behaviours have become regarded as standard practice because of this trend. Indeed, this difficulty might result in an avoidance to confront clients because it involves the risk of falsely accusing someone of being pathological.

### Secret deviant behaviours

Secret deviant refers to exhibiting a *healthy behaviour with an unhealthy mind*. The PTs express that clients can hide their unhealthy behaviours by exhibiting healthy behaviours outwardly, which involve healthy and sound exercise behaviours at the gym and a “normal” body weight. The deviant and unhealthy involves hidden manic minds due to a mental preoccupation with exercise and diet, but also that exercise is performed to avoid anxiety:

Stina: …of course, you cannot let yourself be hypnotized by the healthy masses whom we think of like: God, they are so effective and so consistent at going to the gym and working out. We have actually no clue whether they suffer from it [ON] or not, because we do not know how much it interferes with their lives.

Anna: Yes, exactly.

Stina: It might as well be people who we regard as a normal that have it [ON].

Anna: Yes, absolutely.

Stina: We do not know how much it interferes with their life, there are some things [signs for ill health] that become visible. (FGD 1)

Thus, the PTs emphasize the problematic situation of visibly determining a client’s unhealthy approach because of the hidden manic thoughts. The only possible situation to uncover an unhealthy mind is in a consultation, which they only have with a few people.

In addition, the body size and shape is an important marker for health. As long as clients exhibit healthy behaviours outwardly and have a normal body weight, aspects of the unhealthy approach are perceived to be secretly deviant. In contrast, if an individual exhibits healthy behaviour but has a low body weight, the PTs suspect an unhealthy approach.

### Negotiating between healthy and unhealthy behaviours

Specific sports contexts (*bodybuilding, fitness competitions* and *elite sports*) and groups (*fitness professionals*) in which particular norms rule, influence and complicate PTs’ determination of the border between healthy and unhealthy behaviours. Thus, behaviours that the PTs regarded as ON or as unhealthy in general could at the same time be regarded as conforming and standard practice in these contexts or groups:

Maria: …I barely know where to draw the line between healthy and unhealthy behaviours. I think it is really difficult.

Jesper: Yes, it is.

Mattias: …it is difficult to interpret in general [where to draw the border between healthy and unhealthy behaviours]. If you divide Sweden in three groups; the usual exerciser, the elite exerciser, and those who compete, I think, if I know that [an individual] will compete for something, then I think it is more acceptable that he does follow this more unhealthy behaviour. But then I would ask myself…why is it actually like that? Is it because he will compete and needs to be fully focused? In fact, would it be just as unhealthy if you called yourself a top-level exerciser? Where is the border between there? (FGD 5)

Thus, the quotation highlights the difficulty in determining the border between healthy and unhealthy behaviours. In addition, the PT raises the point about the competition norm when expressing the meaning of the context in classifying the behaviour as healthy or unhealthy. When a competition norm replaces a health norm, the behaviour is regarded as more conforming. Moreover, fitness and bodybuilding competition contexts also legitimate rigid and fanatic dietary and exercise behaviours, since these behaviours are regarded to be aligned with the culture within these sports:

Linnéa: …People doing bodybuilding are really fanatic.

Camilla: Mm.

Linnéa: They [bodybuilders] eat their calories at exact times, and they work out at exact times, they have internal food and sleeping clocks.

Camilla: Ugh, that’s awful.

Linnéa: When they [bodybuilders] can eat normally again, when they finish trying to make weight and stop eating tuna, then their body can’t cope…they feel really bad because they are eating a bowl of pasta or something. There are several that have gone over the edge, but as they have a goal to compete in this [competition], and since no one will react to [their training methods], they must take it to the extreme anyway; it is like the culture in this context. (FGD 3)

As seen in the quote above, the behaviours are considered to be a standard part of preparation before competitions and therefore reasonable. However, the rigid and fanatic behaviours are only conforming in close connection to competitions. If individuals who have competed do not manage to regain “normal” eating and exercise behaviours after competitions, PTs perceive that their behaviours turn into a pathological state. Thus, competition norms only rule in close connection to competition; thereafter, the norm is replaced with a health norm. In addition, the negotiations regarding the border between healthy and unhealthy behaviours, including ON, also occur inside and outside *elite sports* contexts. Behaviours associated with ON and unhealthy behaviours in general outside of elite sports can be regarded as ideal within elite sports. For instance, athletes can exercise every day without being considered to be unhealthy or to have ON. However, PTs perceive that athletes are unhealthy and manic, but legitimate the behaviours since it is a part of their work and they receive a salary for it. Like fitness and bodybuilding competitions, performance and/or competition norms also appear when unhealthy behaviours become conforming in an elite sports context.

Finally, a similar negotiation regarding the border surrounding healthy and unhealthy behaviours occurs when discussing *fitness professionals* (i.e., personal trainers and instructors), since a large amount of exercise is part of their work. For instance, Jonas:We’re excused because we get paid to work out during every other session—it’s accepted. (FGD 4). Thus, a professional fitness norm legitimates unhealthy behaviours among fitness professionals. Although the large amount of exercise is questioned by the PTs, they admit that many fitness professionals perceive that they are “He-men.” This means that many of them perceive that they are invincible and can exercise excessively without it becoming detrimental. The patterns in the negotiations are that professional fitness and performance and/or competitions norms are brought into the fore when legitimizing unhealthy behaviours. In these groups and settings, norms regarding health are replaced, and therefore pure deviant behaviours are not considered pathological.

## Discussion

The findings demonstrated two aspects of ON as pure deviant. On one hand, ON is understood as exhibiting excessive behaviour regarding exercise and eating and, on the other hand, as being in total control. Moreover, ON is conceptualized in terms of unhealthy exercise rather than unhealthy eating behaviours, which differs from previous descriptions (Bratman & Knight, 2000; Bratman, 1997), proposed criteria (Dunn & Bratman, 2016) and the scientific literature (Håman et al., 2015). Thus, it seems that PTs reproduce the general perception of ON in Sweden that, for instance, has been identified in the way Swedish daily newspapers frame ON (Håman et al., 2016). In addition, the emphasis on exercise can also be regarded as natural since PTs practice in a gym context in which exercise is the main focus. However, a clear definition of ON does not yet exist since the scientific body of knowledge regarding ON is still limited (Håman et al., 2015, 2016) and diagnostic criteria have just recently been proposed (Dunn & Bratman, 2016).

These pure deviant behaviours associated with ON break PTs’ health norms. Personal trainers’ health norms include balanced behaviours and practicing behaviours that contribute to well-being, in line with what previously has been identified as central aspects of health norms (Pelters, 2012). The different kinds of deviant behaviours that are expressed by PTs are seen in relation to these health norms, which also constitute the basis for their assessments. Personal trainers are aware that clients can outwardly exhibit balanced behaviours contributing to well-being at the gym. They also confirm that they are impressed by these clients´ behaviours that seem very sound and responsible at a first glance. Personal trainers seem to be influenced by the healthiest view on health characterized by moral obligations and individual responsibility for achieving health (Crawford, ; Lupton, 1995). This view is connected to one of the dominating health discourses in Western societies (Lee & Macdonald, 2010), in which health receives an almost religious status as a main goal in life (Pelters & Wijma, 2016). However, PTs take a highly critical stance on their own reflections regarding their impressions of the clients’ flaunting health behaviours because they are aware that an unhealthy mind can secretly hide behind a healthy appearance. Indeed, their critical stance is essential in order not to encourage the idea that the pursuit of health becomes the only thing that matters in the life of their clients.

The aggressive exercise trend in society, expressed by the PTs, seems to legitimize unhealthy behaviours in the gym by affecting the border and norms for acceptable exercise behaviours. This trend has been noticed and described in the scientific literature (Jönsson, 2009). According to the PTs, these extreme exercise behaviours actually challenge their own health norms, resulting in a sensitive dilemma. Personal trainers express uncertainty about how to approach these clients because they are afraid of falsely accusing someone of being pathological. The PTs encompass contemporary changing ideals regarding the norms of acceptable exercise behaviours. However, norms are subject to constant negotiations and change over time (Becker, 1963). Moreover, certain sport contexts and specific groups are brought to the fore as contributing to the complication of PT negotiations regarding the border between healthy and unhealthy behaviours. In this case, health norms are at stake since people are expected to be more extreme regarding exercise and diet because performance and/or competition norms rule and guide their behaviours. For instance, in bodybuilding and in fitness competitions, as well as in elite sports, more extreme behaviours are standard practice. These extreme behaviours would not be considered acceptable in pursuit of everyday health (cf. Becker, 1963). Regarding fitness professionals, the same pattern is evident. A high amount of exercise in this professional group is justified because it is part of their work. Thus, a fitness profession norm replaces a general health norm (cf. Becker, 1963). The unhealthy behaviours are actually the same inside and outside the various settings and in this specific group, but they are classified differently depending on the norms used to explain the behaviours. In line with this, scholars have asserted that norms and values inside and outside organized sports settings differ (cf., as cited in Tan, Bloodworth, Mcnamee, & Hewitt, 2014). Within formal sports settings (Dale & Landers, 1999; Sundgot-Borgen & Torstveit, 2010) and among competing fitness and bodybuilding participants (Andreasson & Johansson, 2015a), extreme and regulated food and exercise behaviours are standard practice. For instance, among elite athletes in combat sports these behaviours are regarded as part of the sports’ culture (Pettersson, 2013).

The findings indicate that PTs face difficulties in determining the border between healthy and unhealthy behaviours, due to different circumstances and prevailing norms. This uncertainty has also been noted in previous studies among fitness instructors (Bratland-Sanda & Sundgot-Borgen, 2015). In general, PTs have a relatively limited education, and therefore is it reasonable that they experience challenges and uncertainties like these. Moreover, specific knowledge regarding recognition and management of unhealthy behaviours does not seem to be an area of focus in PT schools (Nygård et al., 2015). This might create a difficult situation for the PTs in their professional assessments of determining whether to intervene or not. Indeed, PTs have a great responsibility since they are in a position of power in relation to their clients that in the end might influence the clients’ health.

### Strengths and limitations

Gyms and PTs were selected for the study with the intent of gaining as wide a variation as possible in order to increase transferability (cf. Graneheim & Lundman, 2003). Thus, a strength of this study is the variation of the PTs’ backgrounds and experiences. Another strength is that the analytical process was carried out in several phases. In this process, categories were reflected on and discussed in a systematic manner among the authors, with the second author experienced in the method (cf. Graneheim & Lundman, 2003).

A limitation was the size of some of the FGDs. Nevertheless, a FGD with two participants may also generate, for instance, typical group development stages, although larger focus groups may include more variation (Toner, 2009). Another methodological challenge was related to managing and balancing a critical approach without causing harm (e.g., introducing ON to PTs who have limited knowledge of ON). This was handled by emphasizing that there were no right or wrong answers and that the focus was instead on generating knowledge of how PTs understand and negotiate healthy and unhealthy behaviours in relation to ON. Finally, since this study demonstrates that PT´s emphasize unhealthy exercise in their understandings of ON (i.e., they reproduce Swedish daily newspapers framings of ON), population-based studies might be appropriate for thoroughly investigating this further.

## Conclusion and implications

This study has demonstrated that, in contrast to PTs health norms, ON was regarded as pure deviant and conceptualized in terms of being excessive and in total control regarding eating and exercise behaviours. However, PTs emphasized unhealthy exercise as a main characteristic of ON. Personal trainers maintain that extreme behaviours in the gym are legitimized by an emerging aggressive exercise trend, which contributes to challenge PTs health norms, creating a dilemma in approaching clients and fear of falsely accusing clients of being pathological. Personal trainers assessments of the border between healthy and unhealthy behaviours is complicated since a competition, performance and/or profession norm might prevail.

Thus, the results indicate that a further examination of the learning conditions surrounding PTs might be highly justified, including the contents of PT educations as well as fitness gym policies regarding unhealthy behaviours. Another important topic to address in further research is PTs health promotive efforts and the health advice they offer their clients.

Practical implications include a need to address these difficulties and challenges in PT communities, including formal PT education and continuing professional learning. It might be helpful for PTs to increase their knowledge regarding health promotive efforts and how to detect unhealthy behaviours. However, it is important to emphasize that fitness professionals are not healthcare professionals and not expected to conduct diagnoses. Increased knowledge regarding these topics can nevertheless support their work. Finally, as in Norway, in agreement with Bratland-Sanda and Sundgot-Borgen (2015), it is recommended to propose and implement national official guidelines for identifying and handling unhealthy behaviours in fitness gyms to support PTs and other fitness professionals’ work.
